# Hypothyroidism Enhances Tumor Invasiveness and Metastasis Development

**DOI:** 10.1371/journal.pone.0006428

**Published:** 2009-07-29

**Authors:** Olaia Martínez-Iglesias, Susana García-Silva, Javier Regadera, Ana Aranda

**Affiliations:** 1 Instituto de Investigaciones Biomédicas, Consejo Superior de Investigaciones Científicas y Universidad Autónoma de Madrid, Madrid, Spain; 2 Departamento de Anatomía, Histología y Neurociencia, Facultad de Medicina, Universidad Autónoma de Madrid, Madrid, Spain; Mayo Clinic College of Medicine, United States of America

## Abstract

**Background:**

Whereas there is increasing evidence that loss of expression and/or function of the thyroid hormone receptors (TRs) could result in a selective advantage for tumor development, the relationship between thyroid hormone levels and human cancer is a controversial issue. It has been reported that hypothyroidism might be a possible risk factor for liver and breast cancer in humans, but a lower incidence of breast carcinoma has been also reported in hypothyroid patients

**Methodology/Principal Findings:**

In this work we have analyzed the influence of hypothyroidism on tumor progression and metastasis development using xenografts of parental and TRβ1–expressing human hepatocarcinoma (SK-hep1) and breast cancer cells (MDA-MB-468). In agreement with our previous observations tumor invasiveness and metastasis formation was strongly repressed when TRβ–expressing cells were injected into euthyroid nude mice. Whereas tumor growth was retarded when cells were inoculated into hypothyroid hosts, tumors had a more mesenchymal phenotype, were more invasive and metastatic growth was enhanced. Increased aggressiveness and tumor growth retardation was also observed with parental cells that do not express TRs.

**Conclusions/Significance:**

These results show that changes in the stromal cells secondary to host hypothyroidism can modulate tumor progression and metastatic growth independently of the presence of TRs on the tumor cells. On the other hand, the finding that hypothyroidism can affect differentially tumor growth and invasiveness can contribute to the explanation of the confounding reports on the influence of thyroidal status in human cancer.

## Introduction

The thyroid hormone receptors, encoded by the TRα and TRβ genes, are ligand-dependent transcription factors that belong to the nuclear receptors superfamily [1], [2]. In addition to the well-known role of these receptors in growth, development and metabolism, there is increasing evidence that they have profound effects on cell proliferation and malignant transformation. Reduced expression of TRs as well as alterations in TR genes are common events in many types of human cancer [3]–[14]. In particular, aberrant TRs that act as dominant-negative inhibitors of wild-type TR activity have been found in more than 70% of human hepatocellular carcinomas [15]–[18], and biallelic inactivation of TRβ by promoter methylation as well as mutations in this gene are also frequent in breast cancers [19], [20]. The tendency for TRβ expression to disappear as malignancies progress suggests that TRβ can act as a tumor suppressor in human cancers and that therefore loss of expression and/or function of this receptor could result in a selective advantage for cell transformation and tumor development [21]. We have re-expressed TRβ1 in hepatocarcinoma and breast cancer cell lines that have lost receptor expression and have analyzed the effect of the receptor in tumor progression and metastatic growth. The results obtained demonstrated that TRβ1 expression retards tumor growth, causes partial mesenchymal to epithelial cell transition and has a strong suppressor effect on invasiveness, extravasation and metastasis formation in nude mice [22]. In addition, studies with mice expressing a dominant negative TRβ mutant spontaneously develop metastastic thyroid carcinoma [23] and pituitary tumors [24], and increased aggressiveness of skin tumors is found in genetically modified mice lacking TRs [22], further demonstrating the role of these receptors as inhibitors of tumor progression.

In contrast with the role of TRs as tumor suppressors, no consistent association between thyroidal status and cancer has been demonstrated. For instance, the connection between thyroid disorders and human breast cancer is a controversial issue. Beatson proposed the use of thyroid extracts for breast cancer treatment more than a century ago [25], and hypothyroidism has been described to be frequently found in cancer patients and to be associated with poor response to therapy [26]–[28]. However, a lower incidence of primary breast carcinoma and reduced risk of developing invasive disease have been also reported in hypothyroid patients [29]. Hypothyroidism appears to be clinically favorable in patients with glioblastoma multiforme, since treatment with the anti-thyroidal drug propylthiouracil in combination with tamoxifen appears to increase survival [30]. On the other hand, it has been reported that hypothyroidism might be a possible risk factor for liver cancer in humans [31], and thyroid hormone administration also influences hepatocarcinoma progression in experimental animals. Thus, T3 treatment in rats, despite causing liver hyperplasia, induces a rapid regression of carcinogen-induced hepatic nodules and reduces the incidence of hepatocarcinoma and lung metastasis [32]–[34].

In this work we have examined the effect of hypothyroidism on tumor growth, invasion and formation of metastasis by hepatocarcinoma and breast cancer cells in nude mice. In order to analyze if the changes caused by hypothyroidism are dependent on a direct effect of the hormone in the tumor cell through binding to TRs, we have used both parental SK-hep1 and MDA-MB-468 cells that do not express the receptors and cells in which TRβ1 has been re-expressed [22]. The results obtained demonstrated that hypothyroidism has a dual effect on tumorigenesis. Tumor growth is slower in hypothyroid mice, but the tumors are more aggressive and invasive, and metastasis formation is strongly enhanced. Since these changes are observed in animals inoculated both with parental and TR-expressing cells, they appear to be secondary to changes in the stromal cells as a consequence of host hypothyroidism.

## Results

### Hypothyroidism retards tumor growth

**Figure 1:**
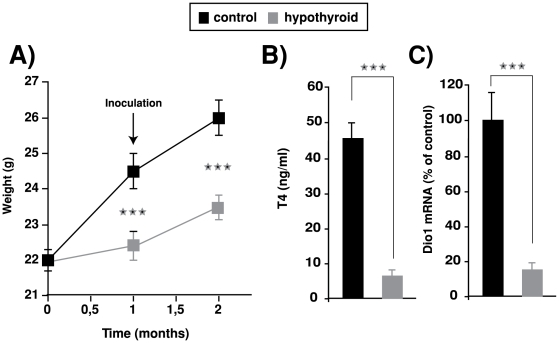
Induction of hypothyroidism. (A)-Weight of control mice and mice treated orally with anti-thyroidal drugs. Mice were treated for 1 month before inoculation of tumor cells. (B)-Circulating levels of T4 measured at the end of the treatment. (C)-mRNA levels of deiodinase 1 (Dio1) in livers from control and hypothyroid mice.

**Figure 2:**
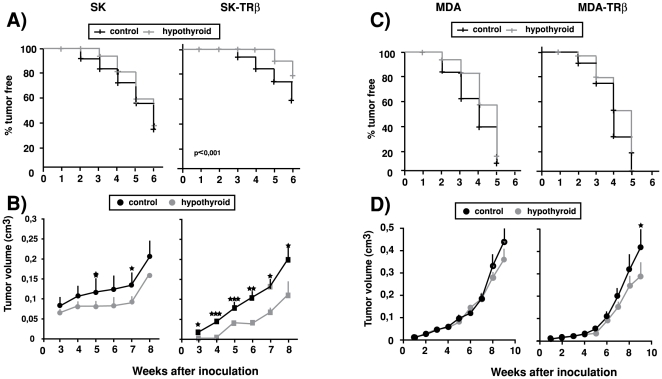
Hypothyroidism retards tumor growth. (A)-Tumor incidence in euthyroid and hypothyroid nude mice injected heterotopically into both flanks with parental (SK) and TRβ1-expressing (SK-TRβ) hepatocarcinoma cells. (B)-Tumor volume was measured at different time points after inoculation in the same experimental groups. (C)-MDA and MDA-TRβ cells were inoculated orthotopically into the mammary fat pad of both groups of mice and tumor appearance was followed. (D)-Tumor volume in xenografts of parental and TRβ-expressing MDA cells. Data are mean±S.E.

Nude mice were made hypothyroid by treatment with anti-thyroidal drugs 4 weeks before inoculation of tumor cells (Figure 1). This treatment significantly retarded the growth of the animals that at the end of the experiments showed a decreased weight in comparison with the untreated controls, strongly reduced the levels of circulating thyroxine, and also markedly decreased transcript levels for deiodinase 1 in liver, a sensitive marker for tissue hypothyroidism [35]. SK and SK-TRβ cells were inoculated subcutaneously into the flanks of control and hypothyroid nude mice and tumor growth was followed (Figure 2). In agreement with our previous observations [22], in euthyroid animals expression of TRβ retarded the detection of palpable tumors (Figure 2A) and significantly reduced tumor volume during the first weeks (Figure 2B). Furthermore, hypothyroidism retarded tumor growth in mice inoculated with both SK and SK-TRβ cells, although the reduction was more marked in the case of the TRβ-expressing cells. When MDA cells were inoculated orthotopically in the mammary gland, tumor appearance was slightly retarded in hypothyroid animals in both parental and TRβ-expressing cells (Figure 2C) and tumor volume was also smaller (Figure 2D), although differences were less marked than those observed with hepatocarcinoma cells and were only statistically significant at 9 weeks post-inoculation of MDA-TRβ cells.

**Figure 3:**
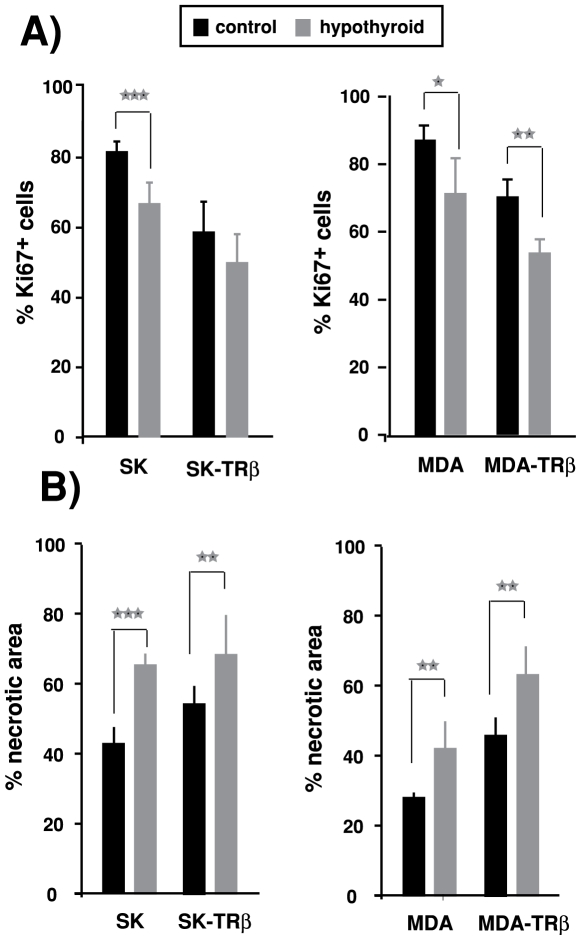
Hypothyroidism reduces tumor proliferation and increases necrosis. (A)-The percentage of cells expressing the proliferation marker Ki67 was determined by immunohistochemistry in biopsies of the tumors formed by parental and TRβ-expressing SK and MDA cells in control and hypothyroid nude mice. (B)-Hypothyroidism increased the necrotic area of the tumors determined from H& staining. Data are mean±S.E.

The reduced tumor volume in hypothyroid hosts correlated with a lower proliferation in tumor biopsies obtained at the end of the experimental period (Figure 3A). Ki67 labeling showed that tumors originated in control mice from parental SK and MDA cells were highly proliferative and that hypothyroidism reduced the number of cells expressing this proliferation marker. In addition, TRβ-expressing cells gave rise to tumors with a lower proliferation index and this reduction was stronger in MDA-TRβ tumors in the hypothyroid mice. Decreased proliferation was accompanied by enlargement of the necrotic area of the tumors grown in hypothyroid mice and, as shown in Figure 3B, this increase occurred both in parental and TRβ-expressing cells.

**Figure 4:**
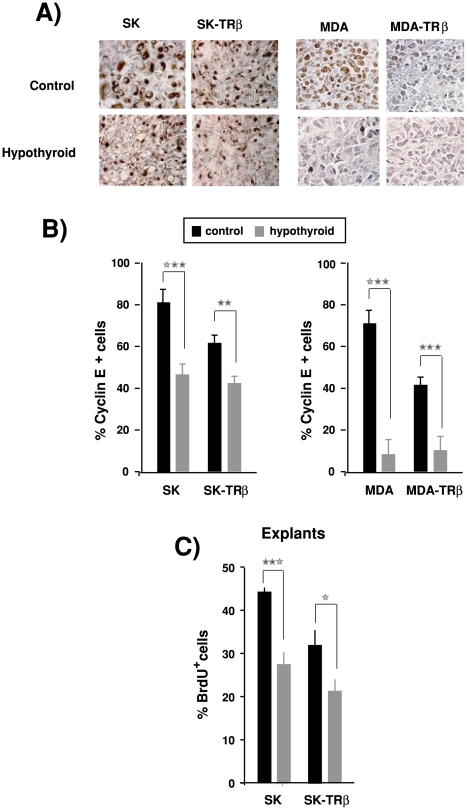
Hypothyroidism reduces Cyclin E expression and BrdU incorporation. (A)-Immunohistochemical staining for Cyclin E in biopsies from SK, SK-TRβ, MDA and MDA-TRβ tumors developed in control and hypothyroid nude mice. (B)-Quantification of the percentage of cells expressing Cyclin E in tumors of the different groups. (C)-BrdU incorporation was measured in explants from SK and SK-TRβ tumors resected after 25 days of inoculation in control and hypothyroid mice.

Expression of Cyclin E, other proliferation marker, was reduced in biopsies of both hepatocarcinoma (Figure 4A) and breast cancer tumors (Figure 4B) developed in hypothyroid hosts, and also in this case the effect was observed independently of the presence of TRβ. In addition, reduced proliferation, assessed by BrdU incorporation, was observed in explants obtained from SK and SK-TRβ tumors when xenografts were grown in hypothyroid hosts (Figure 4C).

### TRβ expression is reduced during tumor growth

**Figure 5:**
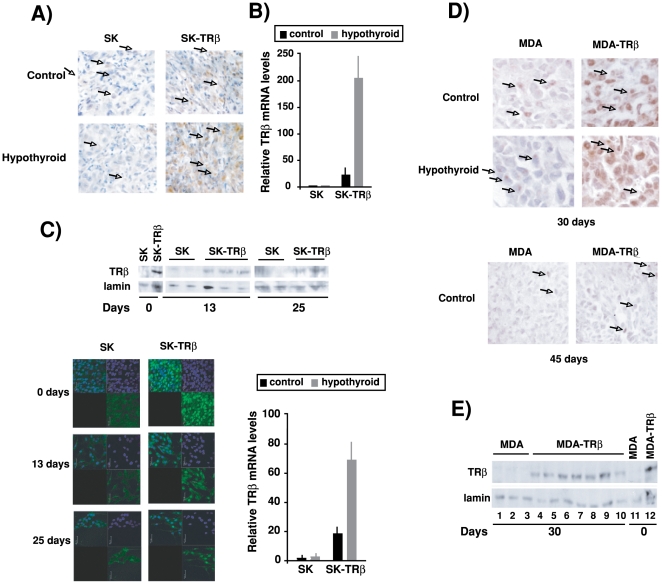
Hypothyroidism prevents TRβ loss during tumor growth. (A)-Immunohistochemical staining for TRβ expression after 30 days of inoculation of SK and SK-TRβ cells in the flanks of control and hypothyroid mice. In the tumors some host inflammatory cells, labeled with arrows, appear to be positive for TRβ. (B)- TRβ mRNA levels analyzed by real time PCR in these tumors. (C)-Detection of TRβ by western blot in explants from individual tumors of SK and SK-TRβ cells obtained 13 and 25 days post-inoculation. Lanes 1 and 2 show TRβ levels of the injected SK and SK-TRβ cells. The lower panels show the immunofluorescence staining for TRβ in the explants. Nuclei were stained with DAPI. (D)-TRβ mRNA levels in explants from SK and SK-TRβ tumors excised 25 days after inoculation of cells in control and hypothyroid mice. (E)-TRβ expression detected by immunohistochemistry in tumors excised after 30 and 45 days of orthotopical inoculation of MDA and MDA-TRβ cells into control and hypothyroid hosts. Inflammatory cells are marked with arrows (F)-Levels of TRβ detected by western blot in the injected cells (day 0) and in explants obtained from MDA and MDA-TRβ tumors at 30 days post-inoculation.

TRβ expression was analyzed by immunohistochemistry in SK and SK-TRβ tumors excised at 30 days post-inoculation. As expected, TRβ was not detected in the parental hepatocarcinoma cells, although it could be detected in infiltrating inflammatory cells from the host. On the other hand, the receptor was present in most cells of the tumors formed by SK-TRβ cells in euthyroid mice and receptor expression appeared to be stronger when tumors were developed in hypothyroid mice (Figure 5A). A similar increase was obtained when TRβ mRNA levels were quantified (Figure 5B). Furthermore, in explants obtained 13 and 25 days after cell implantation a reduction of TRβ expression with respect to the levels present in the inoculated cells was detected by Western blot and by immunofluorescence (Figure 5C). In the explants an increase in TRβ mRNA levels was also observed when the cells were derived from tumors developed in hypothyroid hosts (Figure 5C). Reduction of receptor expression during tumor growth was observed later with MDA-TRβ cells. In sections of MDA-TRβ cell xenografts, immunohistochemistry of TRβ demonstrated expression of the receptor at 30 days post-inoculation and this expression was again stronger in tumors grown in hypothyroid mice (Figure 5D). TRβ expression was confirmed by western blot in tumor explants obtained at this time point (Figure 5E). In contrast, in MDA-TRβ tumors examined at 45 days post-inoculation few positive tumor cells were found (Figure 5D). These results suggest that loss of TRβ expression appear to confer a selective advantage to the hepatocarcinoma and breast cancer cells for tumor growth and that receptor loss is retarded when tumors are developed in hypothyroid hosts.

### Hypothyroidism enhances the mesenchymal phenotype of the tumors

**Figure 6:**
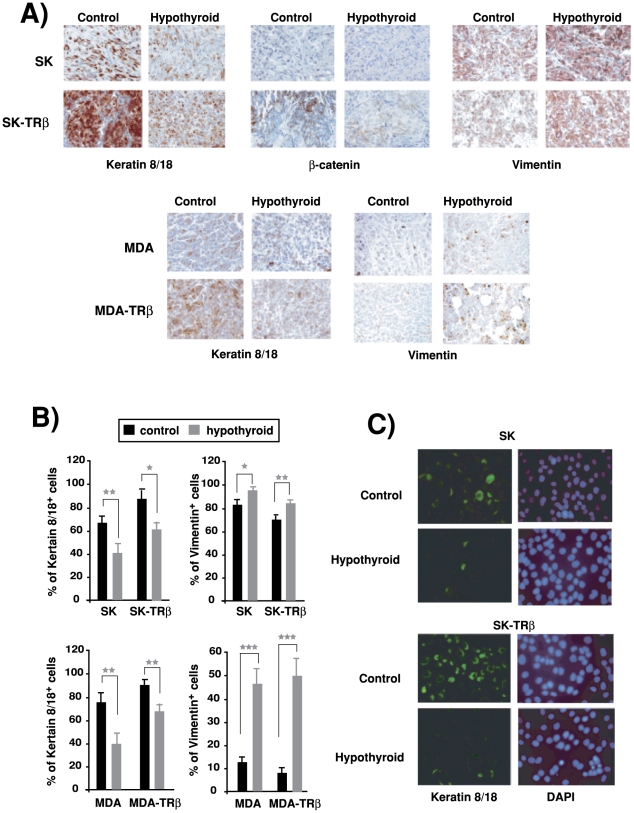
Hypothyroidism enhances the mesenchymal phenotype of tumors. (A)-Immunohistochemical staining for vimentin, cytokeratin 8/18 and β-catenin showed that tumors from inoculated parental SK and MDA cells, as well as from cells expressing TRβ1, showed an increase of the mesenchymal marker and a reduction of epithelial markers. (B)-Quantification of the percentage of cells from the different tumors expressing cytokeratin 8/18 and vimentin. (C)- Cytokeratin 8/18 expression analyzed by immunofluorescence in explants obtained from tumors excised after 25 days of inoculation of SK and SK-TRβ cells into control and hypothyroid mice.

We have previously observed that TRβ1 causes a partial mesenchymal to epithelial transition in the hepatocarcinoma and breast cancer tumors, decreasing the levels of the mesenchymal marker vimentin and increasing the epithelial marker cytokeratin 8/18. Other epithelial marker, β-catenin, was absent in xenografts from MDA cells, but it was expressed in xenografts from TRβ1-expressing SK cells [22]. As shown in Figure 6A, when cells were inoculated in hypothyroid mice, tumors from both parental and TRβ1-expressing SK and MDA cells had a more mesenchymal phenotype with a strong reduction of keratin 8/18 and β-catenin and a concomitant increase in vimentin. Quantification of the percentage of cells positive for these markers confirmed a strong reduction in the expression of the epithelial marker and a significant increase in the expression of the mesenchymal marker in tumors grown in hypothyroid hosts (Figure 6B). In addition, a strong reduction in cytokeratin 8/18 levels was observed by immunofluorescence in explants derived from hepatocarcinoma SK and SK-TRβ tumors originated in the hypothyroid mice (Figure 6C). These results show that hypothyroidism confers a less differentiated phenotype to the tumors independently of the presence of the receptor in the cancer cells.

### Hypothyroidism increases tumor invasiveness

**Figure 7:**
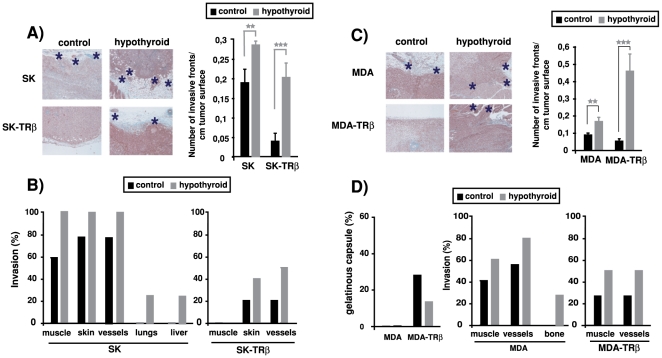
Hypothyroidism enhances tumor invasiveness. (A and C)-Representative H&E staining of tumors formed by parental and TRβ-expressing SK and MDA cells inoculated in control and hypothyroid nude mice (left panels). Tumors originated in hypothyroid hosts were more invasive as illustrated by asterisks that denote sites of tumor invasion. The number of invasion fronts of the tumors was scored and is represented as mean±S.E in the right panels. (B and D)-Quantification of the percentage of animals with tumors infiltrating surrounding tissues such as muscle, blood and lymph vessels and skin or having long distance metastasis in lung, liver or bone. In the case of MDA cells, the number of tumor enveloped by a conspicuous gelatinous capsule was also scored.

Tumors originated from SK cells in normal nude mice are highly infiltrative, presenting an elevated number of invasion fronts. In contrast, tumors from SK-TRβ cells were less infiltrative, presenting a clearly detectable pseudocapsule of collagen and inflammatory cells, and a reduced number of invasion fronts (Figure 7A). This growth pattern was altered when SK-TRβ cells were inoculated into hypothyroid mice, since tumors acquired a more invasive phenotype with a significant increase in the number of invasion fronts. In addition, all hypothyroid animals injected with SK cells had tumors that infiltrated adjacent muscle, lymph and blood vessels or skin (Figure 7B), whereas some of the tumors from euthyroid animals did not invade these tissues. Furthermore, no tumors developed in normal mice caused the appearance of lung or liver metastasis, but such long distance metastasis in these tissues were present in 25% of the hypothyroid animals. In accordance with the less infiltrative pattern of SK-TRβ1 tumors, distant metastasis were not detected in either control or hypothyroid mice and invasion of surrounding tissues was strongly reduced, although it increased in hypothyroid mice (Figure 7B).

Hypothyroidism of the host mice also increased invasiveness of MDA cells. Tumors formed by parental MDA cells in euthyroid mice present a diffuse highly invasive growth pattern, whereas MDA-TRβ cells give rise to tumors with a more compact structure. As in the case of SK-TRβ tumors, they are surrounded by a pseudocapsule and present a reduced number of invasion fronts (Figure 7C). This pattern is lost in MDA-TRβ tumors developed in hypothyroid hosts, where the tumors were more aggressive and the number of invasion fronts increased strongly (Figure 7C). In addition, 30% of the tumors from MDA-TRβ cells are delimited by a macroscopic gelatinous capsule (that is not found in tumors from cells lacking the receptor) and this percentage was reduced to 10% in the hypothyroid mice (Figure 7D). Muscle and vessels infiltration of MDA and MDA-TRβ tumors also increased in the hypothyroid mice, and long distant metastasis in bone were detected in MDA tumors developed in hypothyroid but not in euthyroid animals (Figure 7D).

**Figure 8:**
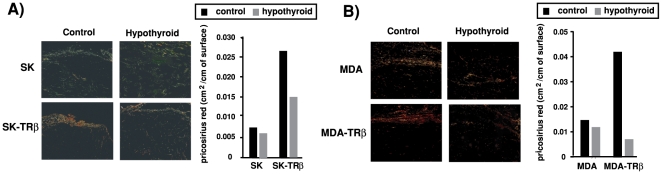
Hypothyroidism causes unpacking of collagen fibers. (A)-Representative images of Picrosirius Red staining (PSR) of collagen in tumors formed by parental and TRβ-expressing SK and MDA cells. Tumors grown in control and hypothyroid mice were examined under polarized light. Red staining characteristic of a well-developed tumor pseudocapsule with ordered collagen was only observed in tumors originating from TRβ-expressing cells inoculated in control hosts. (B)-Quantification of the red-stained areas in the different tumors. Data were calculated as cm^2^ of red staining/cm of tumor surface.

As an additional approach to evaluate changes in tumor infiltration, we also examined connective tissue organization by the Picrosirius red (PSR) staining technique. Under polarized light, the color of PSR staining varies depending on collagen fiber thickness and packing density [36]. Characteristically, red color indicates tightly packed collagen fibers whereas sites of tumor invasion exhibit green birefringence typical of increased extracellular matrix degradation. As shown in Figure 8, red staining characteristic of a well developed collagen pseudocapsule was only clearly detected in tumors formed by TRβ-expressing cells in control mice, whereas green color was predominant in tumors originating from parental cells. Quantification of the area stained in red, demonstrated that the increase observed in the TRβ-expressing tumors was notably reversed when they were developed in hypothyroid hosts, in agreement with the increased invasion found under these conditions.

### Formation of experimental metastasis is enhanced in hypothyroid mice

**Figure 9:**
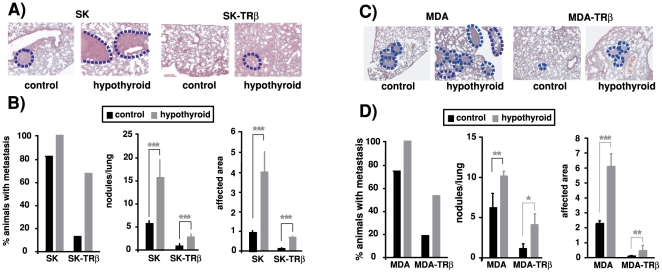
Hypothyroidism enhances formation of experimental lung metastasis. Control and hypothyroid mice were injected with parental and TRβ1-expressing cells into the tail vein and 30 days later lungs were excised mounted and stained. (A and C)-Representative images of lungs from mice injected with MDA, MDA-TRβ, SK and SK-TRβ cells. Metastasis are delineated with a discontinuous blue line. (B and D)-The percentage of animals bearing metastatic lesions, the number of lesions/lung, and the area of lung parenchyma affected (mean±S.E) were determined in the different groups.

The influence of hypothyroidism on formation of experimental metastasis was examined by comparing the appearance of lung metastasis upon inoculation of parental and TRβ-expressing cells into the tail vein of normal nude mice and mice treated previously with anti-thyroidal drugs. In agreement with our previous observations [22], most normal animals injected with parental SK cells developed nodular metastasis in the lungs, whereas less than 20% of the animals injected with SK-TRβ cells developed metastasis (Figure 9A and B). Hypothyroidism significantly increased the incidence of lung metastasis and under these conditions up to 70% of mice inoculated with TRβ-expressing cells had metastatic lesions. The number of metastasis per lung was also strongly enhanced in hypothyroid mice injected with either parental or SK-TRβ cells and the same occurred with the area of the tissue affected (Figure 9B).

Strong enhancement by hypothyroidism of metastasis formation by MDA and MDA-TRβ cells was also observed. When inoculated into euthyroid animals MDA-TRβ cells had a markedly reduced metastatic capacity with respect to the cells that lack the receptor (Figure 9C), and this capacity increased significantly when the cells were inoculated in hypothyroid mice. As in the case of hepatocarcinoma cells, not only incidence of metastasis, but also the number of metastatic lesions and the area of the lung parenchyma affected, was significantly enhanced when either parental or MDA-TRβ cells were injected into the mice treated with the anti-thyroidal drugs (Figure 9D).

## Discussion

There is increasing evidence that reciprocal interactions between the tumor cells and the stromal cells of the tumor microenvironment of the host are critical for tumor progression [37], [38]. In agreement with this idea, this work shows that experimental hypothyroidism in mice has a profound effect on invasiveness and formation of metastasis by hepatocarcinoma and breast cancer cells independently of the cellular expression of TRs.

Epithelial-mesenchymal transition plays a key role in tumor invasion by disrupting intercellular contacts and enhancing motility and migration of tumor cells to the surrounding tissues [39], [40]. We have observed that tumors developed from cells inoculated into hypothyroid nude mice had a more undifferentiated phenotype than those injected into euthyroid mice, as evidenced by enhanced expression of epithelial markers such as keratin 8/18 or β-catenin and by reduced expression of mesenchymal markers such as vimentin. This mesenchymal phenotype can facilitate spreading from the primary tumor to the neighboring host tissues, a critical step that allows tumor cells to invade the extracellular matrix, enter the circulation and disseminate to distant organs.

We have also found that tumors formed in hypothyroid hosts showed changes in the extracellular matrix as demonstrated by a change in the polarization colors of PSR staining from red to green. It is assumed that unpacking of the collagen fibers may facilitate invasion of the surrounding tissues by the tumors, and predominance of green color indicates that the collagen molecules are loosely packed and could be composed of procollagens, intermediates, or pathological collagen rather than tightly packed normal fibers [41], [42]. In accordance with the alteration in the extracellular matrix, as well as with the changes in the tumor cell phenotype, hypothyroidim increased the number of invasion fronts of the tumors and strongly augmented infiltration of adjacent tissues such as muscle, blood and lymph vessels or skin.

Thyroidal status also influenced the formation of long distance metastasis by hepatocarcinoma and breast cancer cells. Thus, spontaneous metastasis in tissues such as lung, liver or bone appeared when cells were injected into hypothyroid but not euthyroid hosts, reinforcing the concept that metastatic growth is dependent on both the intrinsic properties of the tumor cells and the responses of the stromal cells. Furthermore, formation of experimental metastasis in lung by direct inoculation of the cancer cells into the tail vein of the hypothyroid nude mice was also markedly enhanced with respect to the metastatic growth observed in normal hosts. The process of metastasis requires a chain of events (invasion, intravasation, survival in circulation, scattering to distant tissues, extravasation into parenchyma, and colonization of vital organs) that are rate limiting since a failure at any of them can stop the entire process [43], [44]. Therefore, hypothyroidism appears to sustain these different steps and to favor a permissive tissue microenvironment for cancer metastasis.

The observed effects of hypothyroidism on invasion and metastasis could be secondary to the actions of the thyroid hormone on the tumor cell, the host stroma or both. The lower hormone availability in animals treated with anti-thyroidal drugs would decrease hormone binding to the receptors in the tumor cell and host tissues and reduce its biological effects. We have shown that in cultured hepatocarcinoma and breast cancer cells TRβ abolishes anchorage independent growth and migration, and that when inoculated in mice causes partial mesenchymal to epithelial transition and inhibits invasion and metastatic growth [22]. Therefore, a higher aggressiveness of the tumors developed from TRβ-expressing cells in hypothyroid hosts would be compatible with a direct effect on the cancer cells as a consequence of reduced TR activity. Other possibility was that TRβ could be lost selectively when the tumor cells proliferate in hypothyroid mice. That this was not the case was demonstrated by the finding that, although receptor levels were reduced during tumor formation, this loss was even less marked under these conditions. Importantly, we also observed increased malignancy of tumors formed by the parental hepatocarcinoma and breast cancer cells that do not respond to thyroid hormones because they do not express TRs [22]. Therefore, the changes in the stromal cells associated with low thyroid hormone levels rather than a direct effect on the cancer cells appear to be responsible for the increased invasiveness and metastatic activity observed in hypothyroid mice.

A bigger size of the tumors in hypothyroid mice would be compatible with increased cancer cell dissemination and metastatic colonization [45]. However, increased aggressiveness of tumors developed in hypothyroid mice did not correlate with an increase in tumor growth, rather tumors developed faster in euthyroid than in hypothyroid hosts. These results agree with previous results from our laboratory. We have demonstrated that TRs inhibit tumor formation by the *ras*-oncogene in nude mice and that tumor development by *ras*-transformed fibroblasts is retarded in hypothyroid animals [46]. Retardation of tumor growth in hypothyroid mice occurred when both TRβ–expressing cells and cells that do not express the receptor were inoculated, suggesting again that changes in the host stroma associated with hypothyroidism rather than a direct receptor-mediated action on the tumor cells are responsible for inhibition of tumor growth. However, the possibility that “non genomic” actions of thyroid hormones mediated by putative receptors different from TRs [47], [48] could participate in the effect of hypothyroidism on tumor growth, invasiveness and metastasis development cannot be dismissed at present.

In summary, our data point to an important role of the thyroidal status in tumor progression. Normal thyroid hormone levels appear to favor growth of primary xenografts, but they also block tumor cell dissemination and metastasis formation. These divergent effects could help to explain the confounding reports on the influence of hypothyroidism in human tumors. Furthermore, since our results show that similar effects are observed independently of the presence or absence of TR in the cancer cells, it would be expected that thyroidal status could impact tumor progression even in tumors in which TRs are deleted or mutated, a common event in human cancer.

## Materials and Methods

### Ethics Statement

All animal work was done in compliance with the European Community Law (86/609/EEC) and the Spanish law (R.D. 1201/2005(), with approval of the Ethics Committee of the Consejo Superior de Investigaciones Científicas.

### Cell lines

Parental SK-hep1 (SK) and MDA-MB-468 (MDA) cells, and cells expressing TRβ1 (SK-TRβ and MDA-TRβ, respectively), were obtained and grown as previously described [22].

### Xenografts

Groups of athymic nude mice (athymic nude-Nu) 8-10 weeks old were used for xenografting studies. SK and SK-TRβ cells (1×10^6^ cells in 100 µl PBS) were injected subcutaneously into each flank of the mice (5 mice/group) and the same number of MDA and MDA-TRβ cells were inoculated into the fat mammary pad (10 animals/group) as previously described [22]. Similar injections were performed in parallel in normal mice and in mice made hypothyroid by treatment with 0.02% methymazole and 0.1% sodium perchlorate in the drinking water [46]. Treatment started 4 weeks before inoculation and was continued for the duration of the experiments. Tumor volume was measured every week and only tumors with diameter >0,3 cm were considered. The weight of the animals was recorded once a week and at sacrifice tumors were excised, blood was taken for serum measurement of thyroxine (T4) by means of specific radioimmunoassay [49] and samples from different tissues were taken.

### Histology and Immunohistochemistry

Tumors and tissues were processed for histopathologic procedures by fixing in 4% buffered formalin and embedded in paraffin wax. Sections were stained with H&E processed for immunohistochemistry that was performed using standard protocols on deparaffinized sections as previously described [22]. The antibodies used were: TRβ (sc-737; Santa Cruz Biotechnology), cytokeratin 8/18 (NCL-5D3; Novocastra laboratories), vimentin (61013; Progen), β-catenin (610154; Biodiagnostic), Cyclin E (7959 ABCAM), Ki67 (M7240; DakoCytomation). Ki67 was used to determine proliferation index (Ki67-positive cells/total cells) from 5 photographs taken from 4–6 sections of each group (x400). The percentage of cells expressing cyclin E, vimentin or cytokeratin 8/18 was also scored in a similar way. Picrosiruis red (PSR) staining of collagen was performed as described by Junqueira et al. [36]. Stained slides were observed under polarized light using a Leica DMBL light microscope microscope equipped with a polarizer/analyzer set. Quantification of the red staining was performed using analysis@Soft Imaging System. To determine necrotic areas (necrotic cells/total cells), tumors were scanned (Kodak Professional RES 3370) and used for histometric counting. Necrotic cells were expressed relative to total cells. Tumor perimeters were measured from panoramic scannings of Trichromic stained tumors and the number of invasion fronts per cm was scored visually.

### Formation of experimental metastasis

For formation of experimental metastasis in lung, 1×10^6^ cells were injected into the lateral tail vein of control nude mice and of mice treated with the anti-thyroidal drugs for 30 days. Animals were sacrified 30 days after inoculation, and lungs were excised and stained with Masson Trichromic. The number of nodular metastasis was visually scored from scans of the stained tissue, and the total lung areas (x100) and lung areas affected by metastasis (x1000) were calculated using anlySIS ® Soft Imaging System.

### Tumor explants

Tumor explants (2 mm) were prepared from tumors under sterile conditions at different times. Explants were maintained in DMEM∶HAMS (1∶1) with 10% FBS depleted of thyroid hormones.

### Western blot and antibodies

Proteins from cell lysates (20 µg) were separated in SDS-PAGE, transferred to PDVF membranes (Immobilon, Millipore) and used for Western analysis with the anti-TRβ antibody (dilution 1∶500). Lamin, detected with antibody sc-6216 (Santa Cruz Biotechnology, dilution 1∶2000), was used as a loading control.

### Immunofluorescence

Cells from tumor explants were grown on glass coverslips and fixed in a solution containing 4% paraformaldehyde in PBS. Cells were then permeabilized with 0,1% Triton X-100 and after saturation with PBS-0,1 M Glycin were incubated for 2 h with the anti-TRβ antibody (1∶200 dilution) or cytokeratin 8/18 antibody (1∶100). Coverslips were incubated with a fluorescein-tagged secondary antibody (Dakopatts) at 1∶500 dilution and then mounted in Mowiol 4-88 (Hoechst AG). Specimens were observed with an inverted photomicroscope (model DMIRB HC; Leica). Fluorescence images were captured using a cooled digital CCD Hamamatsu ORCA camera and digitally recorded with the ImageProPlus 4.0 imaging software.

### Bromo-deoxi-uridine (BrdU) incorporation

Cells derived from explants, grown on glass coverslips, were incubated 1 h with BrdU. The assays were performed as recommended by the manufacturer (Boheringer Mannheim Biochemica) and cells were stained with DAPI.

### Quantitative real-time PCR assays

Total RNA was extracted using Tri Reagent (Sigma) from the livers of control and hypothyroid nude mice and deiodinase 1 mRNA levels were analyzed by quantitative RT-Q-PCR. RT was performed with 2 µg of RNA following specifications of SuperScript™ First-Strand Synthesis System (Invitrogen Life Technologies). The primers for deiodinase 1 used were: 5′-CTTCGGTGACAGTTTTGATGAGC-3′ (forward) and 5′-GCAACAGATTTGGTGCTGGATG-3. TRβ transcripts were determined in RNA extracted from tumors and explants using the primers: 5′-CCACCT TCTTCAGCATCC-3′ (forward) and 5′-AGTCATCTACGAGTCTCTTG-3′(reverse). PCRs reactions were performed using a MX3005P instrument (Stratagene) and detected with Sybr Green. Data analysis was done using the comparative CT method and data were corrected with the GAPDH mRNA levels.

### Statistical analysis

The Kaplan–Meier method was used to estimate the percentage of tumor free animals, and the Breslow test was used to test for differences between curves using SPSS 12,0. ANOVA analysis was used to evaluate statistical significance in tumor volume curves, number of invasion fronts and necrotic area. Results are expressed as the mean±SE of the indicated number of experiments. The 95% confidence intervals were calculated based on SE of the mean. Statistical significance was estimated with Student's t-test for unpaired observations. In all cases: * p<0.05, **<p0.01, *** p<0.001.
